# A Potentially Advantageous Use of a Zero-Profile, Stand-Alone Anterior Interbody Spacer at C2-3 for the Treatment of Hangman’s Fracture: A Technical Case Report

**DOI:** 10.7759/cureus.16059

**Published:** 2021-06-30

**Authors:** Asad M Ashraf, John K Houten

**Affiliations:** 1 Orthopedic Surgery, Maimonides Medical Center, Brooklyn, USA; 2 Neurosurgery, Hofstra Northwell School of Medicine, New York, USA

**Keywords:** anterior cervical fusion, axis fracture, cervical spine trauma, dysphagia, hangman’s fracture, traumatic spondylolisthesis, zero-profile

## Abstract

Hangman’s fracture or traumatic spondylolisthesis of the axis is a common fracture pattern in the cervical spine. Nonoperative management with an external orthosis is appropriate in select cases. However, when surgery is necessary, both anterior and posterior approaches can be used, and the optimal approach has not been established. Anterior discectomy and fusion with plating at C2-3 may cause dysphagia from plate prominence, while the posterior fusion of C1-3 eliminates motion of an otherwise healthy atlantoaxial joint, resulting in a significant loss of cervical range of motion. We describe the first published application of a stand-alone, zero-profile implant at the C2-3 segment to treat Hangman’s fracture, a technique already successfully used in the C3-7 region for trauma and degenerative applications. A stand-alone, zero profile interbody spacer was employed in anterior C2-3 arthrodesis surgery for Hangman’s fracture in a 61-year-old female following failure of healing after three months in a hard cervical collar. Late postoperative imaging showed successful fusion and the patient had favorable clinical results with relief of neck pain. A zero-profile, stand-alone implant at C2/3 is an attractive option to surgically treat C2 Hangman’s fracture, potentially minimizing dysphagia attributable to an anterior plate and spare the atlantoaxial joint that is fused with C1-3 posterior arthrodesis. The benefits of the application of this technique may be validated with additional studies.

## Introduction

C2 traumatic pedicle or pars fracture, given the eponym “Hangman’s fracture” by Schneider in 1965, is a well-described fracture type of the cervical spine resulting from hyperextension with a secondary component of axial load or rebound flexion [1,2]. Varying degrees of instability are predicted by the degree of fracture displacement and ligamentous disruption, leading to increasing spondylolisthesis of the axis body and separation from the posterior elements [1]. For stable cases, nonsurgical treatment options are often effective with the use of external immobilization in either a hard cervical collar or halo vest orthosis [1]. Unstable fractures demonstrating instability, as determined by C2-3 disc space disruption, prominent C2-3 angulation, or failure to maintain fracture reduction with external immobilization, require surgical fixation, and both anterior and posterior surgical techniques have been described [3].

Although posterior fixation with a direct repair is a successful strategy, it is technically challenging and may not be anatomically possible in patients with small C2 pedicle dimensions or an atypical course of the vertebral artery [4,5]. An alternative, posterior strategy is C1-3 fusion, as the posterior elements of C2 are not attached to the anterior elements and cannot serve as a fixation point, thus necessitating the loss of both the C1-2 and C2-3 motion segments [6]. With C1-2 serving as the most important spinal level for axial rotation of the cervical spine, the impact of fusion at this level on the movement of daily living and quality of life may be very consequential [7].

Anterior cervical discectomy and fusion (ACDF) with an anterior cervical plate has numerous theoretical advantages. The only disc space requiring arthrodesis is C2/3, a segment that has been biomechanically shown to contribute the least to the range of motion (ROM) of the cervical spine [8]. In addition, surgery is among the most commonly performed spinal procedures, and the general approach and technique are very familiar to most surgeons. On the other hand, it has been demonstrated that there is an association between high cervical anterior approaches to the C2/3 and C3/4 levels with postoperative dysphagia [9]. Indeed, dysphagia from the high anterior cervical approach is one of the reasons that there has been reduced enthusiasm in recent years for the use of anterior odontoid screw fixation for type II odontoid fractures in elderly patients [10]. Although a point of some controversy, some authors have found an association of postoperative dysphagia with the use of anterior cervical plates and increasing risk with thicker plate designs [11,12]. Here, we present a novel use of a stand-alone, zero-profile interbody spacer that uses integral fixation without requiring an additional anterior plate to treat Hangman’s fracture which may avoid any dysphagia attributable to a C2-3 anterior cervical plate.

## Case presentation

**Figure 1:**
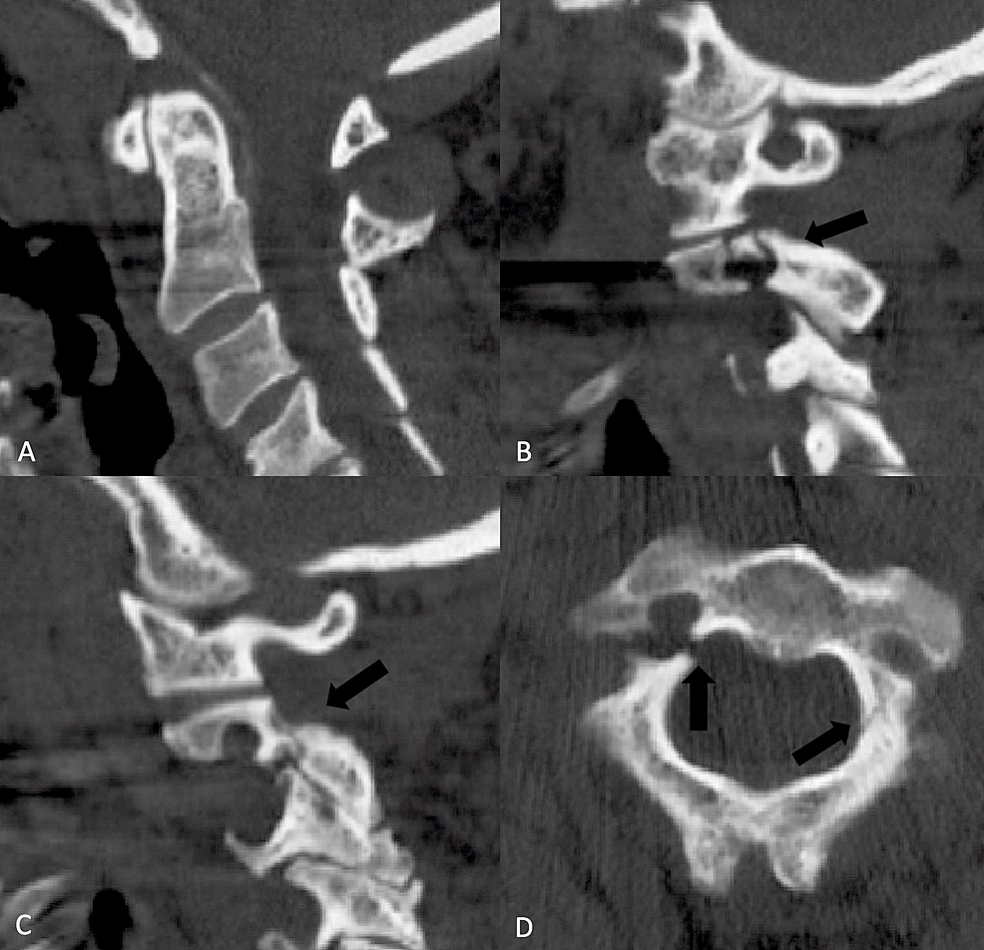
A CT scan of the cervical spine of a healthy, 61-year-old woman following trauma demonstrating fracture though the par interarticularis on the right (B) and left (C). The midsagittal cut (A) showed no evidence of malalignment. Note the very small dimensions of the C2 pedicles seen on the axial image (D) that were judged to be anatomically unsuitable for direct repair surgery. CT: computed tomography

A 61-year-old female with a past medical history of hypertension, emphysema, and diverticulitis presented to the emergency room with neck pain after sustaining a mechanical fall. She fell backward from the stairs and described a flexion-extension “whiplash-type” injury without any recalled blow to the head or the face. She experienced localized pain to the cervical spine without any numbness, weakness, or radicular symptoms. Examination upon arrival to the emergency room demonstrated midline cervical spine tenderness without focal neurologic deficits. A computed tomography (CT) scan of the cervical spine was performed which demonstrated minimally displaced bilateral pars/pedicle fractures of C2 involving the right transverse foramen (Figure 1).

However, a CT angiogram showed no injury to the vertebral arteries. We did not obtain magnetic resonance imaging because of the absence of any signs or symptoms of neurological deficits and the absence of any suggestion of ligamentous injuries on two high-quality CT scans.

**Figure 2:**
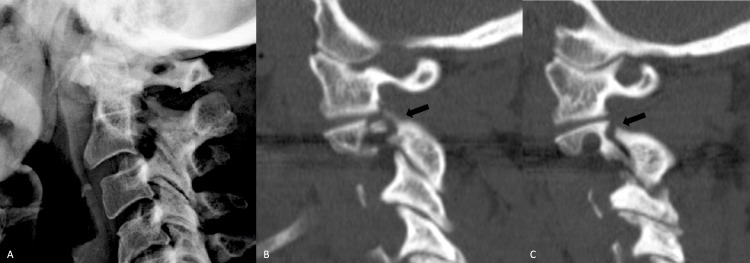
A lateral cervical radiograph (A) two months following the injury shows new anterior displacement of C2 on C3 despite compliance with hard cervical collar immobilization. Sagittal reconstructed paramedian CT images three months following the injury on the right (B) and left (C) show nonunion of the fractures with some widening of the fracture clefts. CT: computed tomography

Due to the minimally displaced nature of the fracture and lack of any neurologic deficit, in addition to the CT finding of extremely small pedicles that were not anatomically suitable for direct repair (Figure 1), she was initially managed nonoperatively with a Miami J cervical collar (Össur Inc., Reykjavik, Iceland), with which we believe she was very compliant. She was followed up with serial X-rays in the course of outpatient follow-up during which she remained neurologically stable but with persisting complaints of neck pain. The two-month post-injury X-ray appeared to show a new 2 mm anterolisthesis of C2/3, and a cervical spine CT scan at three months showed no evidence of bone healing (Figure 2).

She was advised to undergo surgical management of the nonunion given the persisting complaints of pain and the potential for neurological injury from instability. A C2/3 anterior cervical fusion was planned using a zero-profile interbody device with integral fixation.

**Figure 3:**
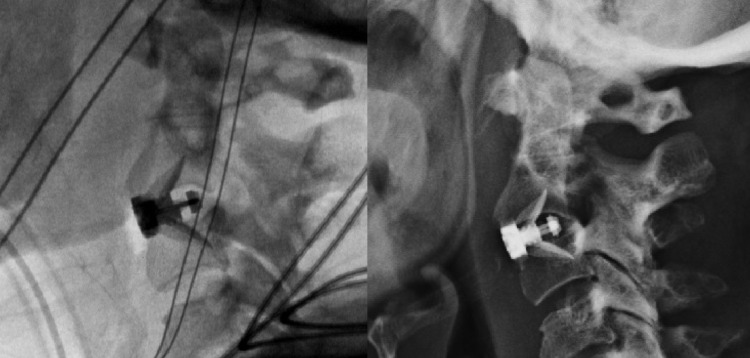
Intraoperative fluoroscopy (left) following the placement of a zero-profile, stand-alone implant at C2/3. The device position was noted to be stable on a six-week postoperative lateral radiograph.

At surgery, the C2/3 anterior exposure was performed using a standard technique with a right-sided transverse incision in a natural skin crease, and intraoperative fluoroscopy verified the appropriate level. The discectomy was performed in a manner to remove the cartilaginous endplates in preparation for the bony endplates while avoiding excessive bone removal that might lead to a risk of subsequent implant subsidence. A 6-mm polyetheretherketone (PEEK) cage (MIS Coalition, Globus Medical, Audubon, Pennsylvania, USA) was packed with demineralized bone matrix (XEMPLIFI, Globus Medical, Audubon, Pennsylvania, USA) and gently tamped until the anterior surface of the cage was aligned flush with the anterior vertebral bodies above and below (Figure 3).

Appropriate positioning was confirmed under fluoroscopy. Two 12 mm integral shims were used to anchor the cage to the vertebra above and below, and the locking mechanisms were engaged. Final imaging with the image intensifier revealed acceptable hardware alignment (Figure 3). There were no operative complications, and the patient was discharged on the first postoperative day.

**Figure 4:**
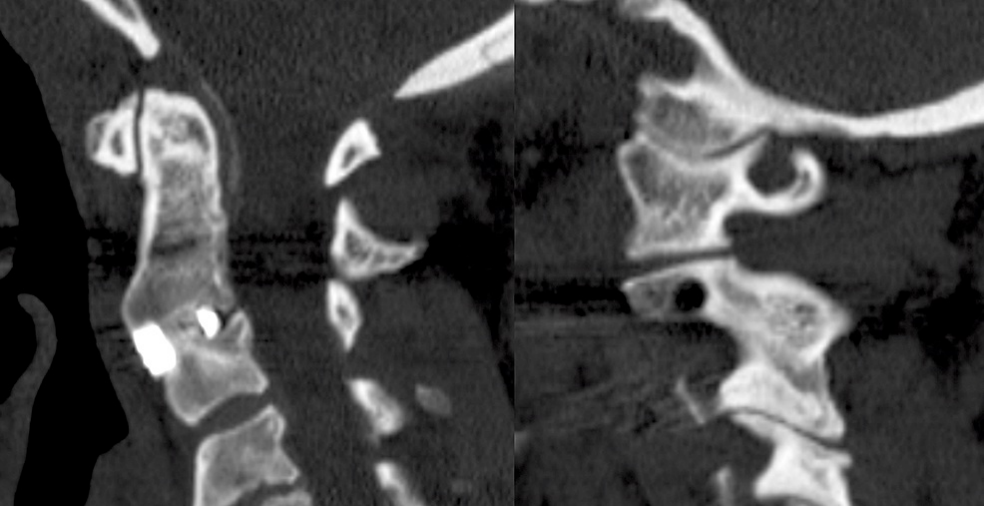
A CT scan midline sagittal reconstructed image at one-year post-surgery showing a solid arthrodesis across the C2/3 disc space (left). Interestingly, a parasagittal image shows that the fracture line has also healed and the facet joint has fused, presumably because of the immobilization of the spinal segment resulting from the anterior fusion. CT: computed tomography

Following surgery, she was maintained in a hard cervical collar for 3 months. She complained of moderate dysphagia and hoarseness at the two-week follow-up that had resolved at the one-month follow-up visit, at which time she also noted resolution of the preoperative neck pain. Serial X-rays of the cervical spine revealed no change in alignment or loss of implant fixation (Figure 3). At one-year post-surgery, a CT of the cervical spine showed evidence of a solid C2/3 arthrodesis (Figure 4).

## Discussion

Posterior-approach surgical management of traumatic spondylolisthesis, commonly known as Hangman’s fracture, is made more difficult by the anatomical nature of the injury which dissociates the posterior elements of C2 from the vertebral body, making stabilization necessary to span from C1-3 when direct repair techniques are judged to be unsuitable. This is disadvantageous in that it fuses the C1/2 segment, the most important in the cervical spine with regard to axial rotation and a joint that is usually uninjured and healthy. C2/3 ACDF is an effective anterior-approach alternative that spares the C1/2 segment but has the shortcoming of potentially causing dysphagia, an issue that has been suggested to be more problematic with increasing thickness of anterior cervical plates [12]. For example, Wang et al. reported a significant decrease in dysphagia rates for patients undergoing ACDF with a stand-alone interbody spacer compared to traditional plate fixation [13].

Stand-alone, zero-profile interbody spacers, utilizing integral fixation with either screws or shims that extend from the spacer and enter the vertebral body through the endplate, avoid the use of a separate anterior plate. Njoku et al. published a series of 41 patients in whom ACDF was performed at the C3-T1 levels using a PEEK interbody cage with an integral titanium screw fixation mechanism that resulted in favorable clinical and radiological fusion outcomes comparable with traditional techniques for ACDF with a separate plate and screws [14]. Brembilla et al. reported the successful use of zero-profile devices in the single-surgery treatment of traumatic injuries, though the study only included injuries involving the C3-7 levels [15]. Kim et al. published a series of 17 patients with traumatic disc injuries from C3-7 and encountered favorable clinical results comparable to those achieved in the treatment of conventional spondylosis with ACDF using a separate anterior plate [16].

In principle, a C2/3 anterior fusion with a stand-alone, zero-profile device in the treatment of Hangman’s fracture is attractive in that it fuses only the C2/3 space while minimizing any dysphagia that might be attributable to the prominence of an anterior plate [17].

Avoiding the prominence of an anterior plate may be of benefit, as meta-analyses performed by Xiao et al. and Yang et al. found decreased rates of postoperative dysphagia in zero-profile spacer cases compared to plate and cage constructs [17,18]. These findings, however, are not universally accepted and are contradicted by Fisahn et al. who found no statistically significant difference in dysphagia between zero-profile and plate fixation [19].

This application of a stand-alone spacer is subject to the shortcomings and limitations of the technique compared with anterior fusion with a plate that have previously been described, including a potentially increased chance of implant subsidence, pseudoarthrosis, and loss of sagittal alignment [14,16]. In addition, there is a concern that trauma may be different from degenerative disease in that the former may be associated with greater potential instability that a stand-alone device would need to overcome. Jumped facets and other injuries that are associated with extensive ligamentous disruption and instability may be less suitable for a stand-alone, zero-profile fixation than for Hangman’s fracture. However, Njoku et al. and Kim et al. noted that biomechanical studies appear to support the suitability of the technique even for traumatic indications and that the theoretical concerns regarding implant fixation strength have not manifested in any unfavorable clinical outcomes [14,16].

We observe that the prevertebral soft tissue shadow on a lateral cervical radiograph is considerably smaller in the C2/3 region compared with the midcervical or lower cervical region, a finding noted in the radiology and emergency medicine literature [20]. Thus, we posit that even if plate thickness has limited or no effect upon dysphagia in the more commonly treated locations of the anterior cervical spine, an effect may be present at C2/3 where the normal soft tissue shadow is only a few millimeters. Therefore, we believe that the technical note in this communication describing the first published use of a zero-profile device for anterior approach fusion to treat Hangman’s fracture is worthy of clinical consideration and further investigation.

## Conclusions

Surgical management of C2 Hangman’s fracture with ACDF using a stand-alone, zero-profile interbody device is a successful surgical strategy that offers an attractive alternative to C1-3 posterior fusion that spares the atlantoaxial joint. Moreover, the technique may avoid dysphagia attributable to anterior plate thickness with a C2/3 ACDF. Additional investigation of the specific application of a stand-alone, zero-profile device for Hangman’s fracture is merited, and larger studies are needed to validate the technique’s potential clinical benefits.
